# Prozone effect of serum IgE levels in a case of plasma cell leukemia

**DOI:** 10.1186/1756-8722-3-32

**Published:** 2010-09-10

**Authors:** Giampaolo Talamo, William Castellani, Nathan G Dolloff

**Affiliations:** 1 Penn State Hershey Cancer Institute, 500 University Drive, Hershey, PA 17033 USA

## Abstract

We describe a case of multiple myeloma (MM) and secondary plasma cell leukemia (PCL) secreting IgE-kappa immunoglobulin. To our knowledge, only 2 cases of IgE-producing secondary PCL have been reported in the medical literature. In our patient, the only tumor marker available for monitoring the therapeutic response to chemotherapy and allogeneic stem cell transplantation was the quantitative M component at serum protein electrophoresis (SPEP), because serum free light chains were in the normal range, Bence-Jones proteinuria was absent, and quantitative serum IgE levels provided inaccurate and erratic results, due to the prozone effect. This is a laboratory phenomenon that occurs when antigen excess interferes with antibody-based methods requiring immune complex formation for detection. It is important to recognize the presence of a prozone effect, because it can produce falsely normal results, and therefore it could lead clinicians to incorrect assessment of the response to therapy.

## Background

IgE myeloma is a very rare subtype of MM, and it represents < 0.01% of all plasma cell dyscrasias [1]. Since the first case was described in 1967 [2], approximately 47 cases of IgE MM have been reported in the literature [3-6]. IgE antibodies are named from the ragweed E antigen, which was used for their isolation, and they are involved in allergic responses, atopic conditions, helminthic and respiratory infections, and chronic inflammatory diseases [7]. It is important to note that commonly available serum immunofixation (IFE) testing screens only for monoclonal IgG, IgM, and IgA chains. Therefore, IFE specific for IgD and IgE should be requested when these rare subtypes are suspected (e.g., when a monoclonal protein has been detected by SPEP, but routine IFE is negative). The clinical manifestations of IgE MM are similar to those seen in other MM subtypes, but some experts consider IgE MM an aggressive disease, associated with a significantly higher rate of plasma cell leukemia [8,9]. Other data do not support the aggressive nature of this subtype of MM. A review of the first 19 reported cases of IgE MM showed no difference in the incidence of extramedullary plasma cell infiltration compared with other subtypes of the disease [10].

We describe a case of IgE-kappa MM and secondary PCL with falsely normal serum levels of IgE due to the prozone effect.

## Case Presentation

**Figure 1:**
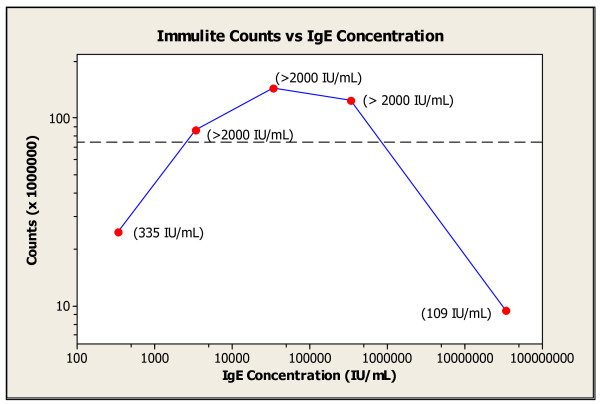
**Immulite^® ^readings of multiple serial dilutions of the same sample showing prozone effect**. The calculated concentration of IgE based on the final measured reading times the dilution factor is plotted along the X axis against the actual instrument reading (counts per second) along the Y axis. Both axes are logarithmic and the dilutions that were used were 1:100,000, 1:10,000, 1:1000, 1:100, and undiluted. In parenthesis at each dilution point is the reading reported by the instrument. The dotted line represents the highest reported value of 2000 IU/mL. The lines connecting each point are for illustration and do not represent the actual values at intermediate dilutions.

A 53 year-old Caucasian man with unremarkable past medical history was diagnosed with MM in November of 2006. He presented with back pain, and MRI of the spine revealed multiple compression fractures. Skeletal survey was negative for lytic lesions. Bone marrow aspirate revealed 75% kappa-restricted atypical plasma cells, establishing the diagnosis of MM. Cytogenetic analysis was normal, and the translocation t(11;14) was the only abnormality detected by the MM FISH panel. IFE was positive for monoclonal IgE-kappa proteins, IgE level was 5,300,000 IU/mL, serum free kappa was normal, and Bence-Jones proteinuria was absent. Patient received treatment with multiple regimens, which included dexamethasone, thalidomide, bortezomib, and lenalidomide. However, 28 months after the diagnosis, MM became refractory to those agents, and patient was referred to our Institution for autologous stem cell transplantation. Our review of the peripheral smear showed circulating atypical plasma cells, representing 52% of the WBC (12,600/μL), and we made the diagnosis of secondary PCL. Bone marrow aspirate contained 80% plasma cells, harboring the original cytogenetic features. At flow cytometry, these cells were positive for CD38, CD138, and negative for CD56 and CD20. Initially, serum level of IgE was reported as normal, but a distinct M peak was present on SPEP. The result of the IgE level was found to be falsely normal due to the "prozone effect". Our laboratory observed the paradoxical increase of the IgE levels with progressively increasing dilutions of the serum sample (Figure 1). Capillary zone electrophoresis for SPEP and serum immunotyping was performed by the Capillarys 2 capillary method (Sebia Electrophoresis, Norcross GA). Serum IgE levels were measured by the Siemens Immulite 2000^® ^(Flanders NJ), using the Total IgE method. All serum IgE dilutions were performed manually, using the manufacturer's diluent.

In view of the plasma cell leukemia, we elected to proceed with an allogeneic instead of autologous transplantation. After induction therapy with 2 cycles of VDT-PACE (bortezomib, dexamethasone, cisplatin, doxorubicin, cyclophosphamide, and etoposide, given at the doses and schedule described elsewhere [11]), patient underwent a non-myeloablative allogeneic stem cell transplantation from his HLA-identical sister, using fludarabine and cyclophosphamide as conditioning regimen. The post-transplant evaluation at day 100 revealed full hematologic recovery, absence of circulating plasma cells in the peripheral blood -even by flow cytometry-, no evidence of graft-vs-host-disease, and MM in partial remission by serum M component and bone marrow biopsy. Monitoring of disease response during the treatment was based on the quantification of the serum M at SPEP, because IgE levels were found to be inaccurate and erratic (Table 1).

**Table 1:** Erratic serum levels of IgE during the response of MM/PCL after allogeneic stem cell transplantation.

DAY	Serum M component (g/dL)	Serum IgE (I.U./mL)
-64	1.6	19,280,000

-48	1.3	---

-29	1.2	14,110,000

-8	0.4	14,233,000

+15	0.4	115,000,000

+21	0.4	---

+35	0.4	---

+49	0.4	2,910,000

+89	0.4	23,700,000

Day -64 is the first day of induction chemotherapy, and day 0 is the day of allogeneic stem cell transplantation.

## Conclusions

PCL is distinguished in "primary PCL", which occurs as a de novo presentation of the leukemia, and "secondary PCL", which is the leukemic transformation of a previously diagnosed MM. Our patient had the secondary form, because it developed 28 months after the initial diagnosis of MM. To our knowledge, 8 other cases of IgE-producing PCL have been reported in the medical literature, and only 2 of them were secondary PCL [12,13]. The incidence of high-risk chromosomal abnormalities, such as complex karyotype and monosomy 13, is high in patients with secondary PCL [14]. However, in our patient, malignant plasma cells both in peripheral blood and BM displayed the same cytogenetic abnormalities observed at baseline, i.e., only the translocation t(11;14)(q13;q32) at FISH, and no other chromosomal aberrations. Of note, the t(11;14) translocation is considered a hallmark of IgE, IgM, and nonsecretory MM, all rare subtypes of MM [15]. Interestingly, a recent publication described a case of IgE MM associated with very high serum levels of serum CA125 (1292.3 U/mL) [16], a tumor marker expressed in various cancers, including ovarian carcinoma and hematologic malignancies [17]. We did not confirm this association in our patient, because his serum CA125 level before induction therapy was 17.9 U/mL, within normal limits (0-34 U/mL).

An important aspect of our case was the unreliability of quantitative IgE levels in the assessment of disease response, due to the prozone effect. Response to therapy in our case was best monitored with the quantification of the M component at SPEP. The recent introduction of the quantitative serum free light chains (FLC) assay has offered another useful tumor marker for monitoring response to therapy in MM [18]. Due to the rarity of IgE MM, no sufficient data of the use of FLC in this subtype of MM are available. In our patient, the serum FLC assay had no role in assessing response to therapy, because the serum free kappa level was always within normal limits.

The prozone effect is a laboratory phenomenon that occurs when antigen excess interferes with antibody-based methods requiring immune complex formation for detection. For immunometric immunoassays, detection of the analyte (in this case, serum IgE) requires that each molecule binds to two separate reagent antibodies in an antibody-analyte-antibody complex: one antibody that "captures" the antigen and the second that provides a detection signal. With excessive amounts of analyte, each reagent antibody binds to separate analyte molecules, not forming the complex essential for detection. The presence of this "high-dose hook" effect should be suspected when the result on a diluted sample is higher than in the undiluted sample. The prozone effect is a well know phenomenon that may complicate the interpretation of various quantitative assays, including those for IgG and IgA [19,20], and laboratory protocols to avoid it have been proposed [19]. The elevations that produce such results are so high that significant manual dilutions are required to bring the concentration into the reporting range of the instrument, and dilution errors are common. For this patient, a 1:10,000 or 1:100,000 dilution was required to obtain a reading, a difficult task even for experienced bench personnel. The variability in serum IgE levels shown in Table 1 may be explained by the challenge of diluting each sample a minimum of 10,000 fold, when standard medical laboratory techniques rarely require a dilution greater 1:100. It is important to recognize the presence of a prozone effect, because it can produce falsely normal results. Due to this effect, the use of only IgE levels for monitoring the response to therapy in our patient could have led the clinicians to inappropriate interpretations of the results and possible therapeutic mismanagement.

## Competing interests

The authors declare that they have no competing interests.

## Authors' contributions

GT was responsible of the patient's treatment and conceived the study. WC carried out acquisition of data, laboratory analyses, and their critical interpretations. ND coordinated the study and helped to draft the manuscript. All authors read and approved the final manuscript.

## Consent

Written informed consent was obtained from the patient for publication of this case report. A copy of the written consent is available for review by the Editor-in-Chief of this journal.
